# Health education technologies for people with disabilities: scope review

**DOI:** 10.1590/1518-8345.8054.4861

**Published:** 2026-07-20

**Authors:** Danielle Figueiredo Patrício, Alexsandro Silva Coura, Maria do Socorro Alécio Barbosa, Tereza Natália Bezerra de Lima, Morgana Cristina Leôncio de Lima, Kelly Cristina do Nascimento

**Affiliations:** 1 Universidade Estadual da Paraíba, Departamento de Enfermagem, Campina Grande, PB, Brazil.; 2 Scholarship holder at the Fundação de Apoio à Pesquisa do Estado da Paraíba (FAPESQ), Brazil.; 3 Universidade de Pernambuco, Departamento de Enfermagem, Recife, PE, Brazil.; 4 Universidade Federal da Paraíba, Departamento de Enfermagem, João Pessoa, PB, Brazil.

**Keywords:** Educational Technology, Assistive Technology, Health Education, Person with Disability, Nursing, Revision.

## Abstract

**Objective:**

to map educational technologies developed and applied in health education for people with disabilities.

**Method:**

scope review based on the recommendations JBI, using the following inclusion criteria: addressing the topic under study; text available in full and written or translated into Portuguese, English, and/or Spanish. Four information sources and grey literature were consulted, in January 2024.

**Results:**

the review covered eight studies, that addressed experiences with planning, construction, evaluation, and validation of technologies aimed at the health education process for people with disabilities and the professionals involved in their care.

**Conclusion:**

educational technologies in health for people with disabilities constitute tools for promoting inclusion and autonomy. The predominance of methodological studies reveals gaps in effectiveness analysis and the need for more robust and diversified research.

## Highlights

**(1)** Educational technologies are relevant tools for people with disabilities. **(2)** They contribute to promoting the inclusion and autonomy of people with disabilities. **(3)** They have good acceptance, accessibility, and potential for inclusion, especially in nursing practice. **(4)** There is a predominance of methodological studies and a need for effectiveness research.

## Introduction

People with disabilities are defined by the World Health Organization (WHO) as those who have long-term physical, mental, intellectual, or sensory impairments that, in interaction with social, attitudinal, and environmental barriers, may hinder their full and effective participation in society on an equal basis with others^1^. It is estimated that more than one billion people worldwide live with some form of disability, a number that could reach two billion by 2050 because of population aging and the higher prevalence of chronic disabling diseases. In Brazil, data from the 2022 Census indicate that 18.6 million people aged two years or older have some disability, representing 8.9% of the Brazilian population in this age group^1^ ^-^ ^2^.

The recognition of disability as a human rights issue was consolidated in the 1948 Universal Declaration of Human Rights and reaffirmed in the Convention on the Rights of Persons with Disabilities^3^. These documents establish universal principles of dignity, equity, and social participation, requiring states to ensure that public policies promote accessibility, inclusion, and autonomy. This paradigm shift moves the understanding of disability from a biomedical model to a social approach, focused on eliminating barriers and valuing individuals’ abilities^4^ ^-^ ^5^.

In this context, Assistive Technologies (AT) are understood as resources, strategies, products, and services that aim to expand the functional abilities and social participation of people with disabilities^6^ ^-^ ^7^. They range from orthotics and prosthetics to screen-reading software, alternative communication systems, and architectural adaptations, constituting fundamental tools for autonomy and inclusion. At the same time, Educational Technologies (ET), whether printed, digital, or hybrid, have proven to be central to the teaching-learning process in health, enabling the mediation of knowledge and the construction of more accessible and participatory practices^8^ ^-^ ^9^.

The integration of AT and ET addresses practical challenges faced by people with diverse disabilities. Deaf people, for example, face communication barriers in health services, which can be minimized by *Libras* applications and bilingual materials^10^. Individuals with intellectual disabilities can benefit from digital educational games and simplified visual resources in the health education process^11^. Similarly, exergames have been used in the rehabilitation of individuals with physical or cognitive disabilities, promoting functional and social gains through playful activities^12^. Population aging and the increase in chronic diseases also expand the number of people who acquire disabilities throughout their lives, making the need for comprehensive care and rehabilitation actions urgent^13^. Beyond the clinical dimension, the health education process is a strategic tool, as it promotes not only the transmission of information but also the development of critical awareness, thereby fostering autonomy, protagonism, and social transformation^14^ ^-^ ^15^.

Meanwhile, the link between assistive technologies and health education is fundamental to increasing the autonomy and inclusion of people with disabilities. Assistive technologies, by providing resources that promote functional independence and social participation, serve as strategic tools when integrated into educational processes, thereby enhancing access to information and supporting health promotion. In this scenario, nursing plays a central role due to its continuous proximity to users, mediating the use of these technologies, translating information into accessible language, and encouraging individuals to take a leading role in their self-care. This articulation not only improves clinical and rehabilitation outcomes but also strengthens the social and civic dimensions of care^5^ ^-^ ^6^ ^,^ ^11^.

In this context, ET emerge as essential resources in health teaching and learning. They can take different forms: assistive technologies (resources that promote functionality and autonomy, such as prostheses, reading software, and physical adaptations); traditional educational technologies (primers, guides, manuals, printed games); and innovative digital technologies, such as exergames and interactive applications, which integrate playfulness, physical activity, and learning^7^ ^-^ ^8^ ^,^ ^16^. All of them share the potential to transform the educational process into a dialogical and participatory space, thereby strengthening citizenship and social inclusion^17^ ^-^ ^20^.

Health education practices, when directed at people with disabilities, should combine direct and indirect care, integrating physical, subjective, and social dimensions. Nursing professionals, due to their close and continuous involvement, play a central role in this process, both in rehabilitation and in promoting inclusion and empowerment, moving beyond the curative model and recognizing people with disabilities as active subjects of care^21^ ^-^ ^22^. 

Given this scenario, this study aims to map the educational technologies developed and applied in health education for people with disabilities.

## Method

This scoping review enabled evidence mapping by exploring the breadth and extent of the literature, to identify gaps and suggest future studies^23^. A scoping review is a type of research that provides an overview of the number and characteristics of available studies, intending to identify the nature and extent of scientific production^24^.

This research was based on the JBI scope review guide, which highlights the steps in the construction process: 1) Define and align the objectives and research question; 2) Develop and align the inclusion criteria with the objectives and research question; 3) Describe the planned approach to identifying the evidence; 4) Search for evidence; 5) Select the evidence; 6) Extract the data; 7) Present the results in graphs and tables; 8) Summarize the findings in relation to the objectives and research question^23^. The protocol for this study is published on the Open Science Framework platform: https://osf.io/a547g/https://osf.io/a547g/.

### Identification of the research question

The PICo strategy was used to develop the guiding question and outline the search. This strategy includes the following elements summarized in the acronym: P = Problem (Health Education); I = Interest (Educational Technology) and Co = Context (People with Disabilities). Therefore, the guiding question of this research is: what educational technologies are used to promote health education among people with disabilities?

### Search strategy

Among the virtual portals available for data collection, the following databases were selected: Medical Literature Analysis and Retrieval System Online (MEDLINE), Excerpta Medica database (Embase), Virtual Health Library (VHL), Latin American and Caribbean Health Sciences Literature (LILACS), and Google Scholar (for gray literature) were selected because they are widely recognized in the field of health. The objective was to analyze scientific production on the proposed theme in national and international contexts.

The advanced search for articles was conducted in January 2024, simultaneously in the five databases. The selection and evaluation of studies were conducted independently and blinded by two reviewers, and any disagreements were resolved by consensus with a third reviewer^25^. To select the initial studies and exclude duplicates, the reviewers read the titles and abstracts using the Rayyan online reference manager. The selections made by each reviewer were systematically sorted using the software and then compared with one another to validate the final sample^26^.

The keywords were selected from the Health Sciences Descriptors (DeCS), namely: “Educational Technology,” “Disabled Persons,” “Health Education,” using the Boolean operators OR and AND. They were combined in different ways, such as: *Educational Technology OR Health Education AND Persons with Disabilities*; *Educational Technology AND Health Education AND Persons with Disabilities*, in Portuguese and English. The results are shown in Figure 1 below:

**Figure 1:** Table describing Boolean operators for database searches. Campina Grande, PB, Brazil, 2024

Search mechanism	Search terms	Quotes retrieved
MEDLINE	*(“Educational Technology”\[MeSH Terms] OR “educational technology”\[tiab] OR “tecnologia educacional”\[tiab]) AND (“Health Education”\[MeSH Terms] OR “health education”\[tiab] OR “educação em saúde”\[tiab]) AND (“Disabled Persons”\[MeSH Terms] OR “people with disabilities”\[tiab] OR “pessoa com deficiência”\[tiab])*	78
Embase	*(‘educational technology’/exp OR ‘educational technology’\:ti,ab OR ‘tecnologia educacional’\:ti,ab) AND (‘health education’/exp OR ‘health education’\:ti,ab OR ‘educação em saúde’\:ti,ab) AND (‘disabled person’/exp OR ‘people with disabilities’\:ti,ab OR ‘pessoa com deficiência’\:ti,ab)*	57
VHL*	*(“Tecnologia Educacional” OR “tecnologia educacional” OR “Educational Technology”) AND (“Educação em Saúde” OR “Health Education”) AND (“Pessoas com Deficiência” OR “pessoa com deficiência” OR “People with Disabilities”)*	23
LILACS	*(“Tecnologia Educacional” OR “Educational Technology”) AND (“Educação em Saúde” OR “Health Education”) AND (“Pessoas com Deficiência” OR “People with Disabilities”)*	29
Gray literature	*(“tecnologia educacional” OR “educational technology”) AND (“educação em saúde” OR “health education”) AND (“pessoa com deficiência” OR “people with disabilities”)*	13
**Total**		200

*VHL = Virtual Health Library

### Eligibility criteria

The selection of studies analyzed was based on the following inclusion criteria: addressing the topic under study; full text available and written or translated into Portuguese, English, and/or Spanish. The exclusion criteria were: incomplete texts or texts that were unavailable; repetition of the same article in more than one database; and lack of relevance to the subject under study. No time frame was set for the search to retrieve as many studies as possible.

### Data processing and analysis

**Figure 2:**
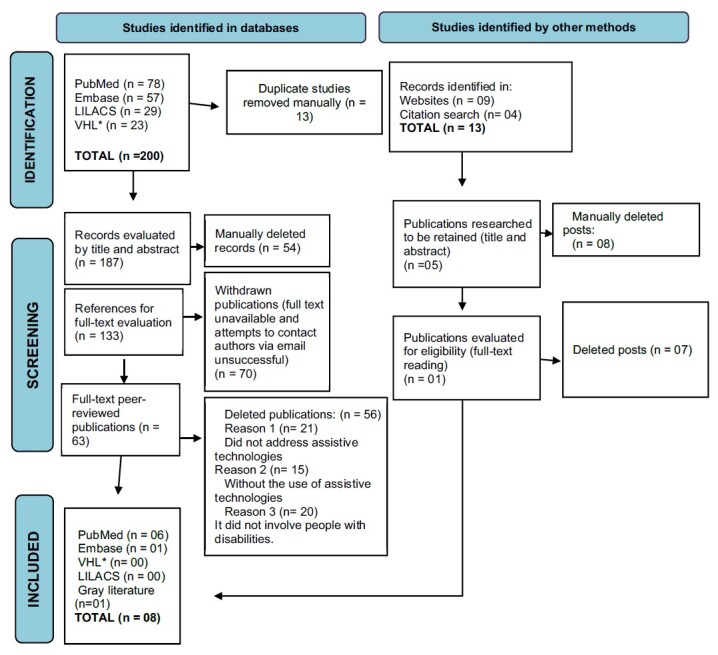
PRISMA-ScR flowchart used for study identification and selection. Campina Grande, PB, Brazil, 2024

The PRISMA-ScR guidelines for scoping reviews^27^ were used to graphically represent the study screening procedure, as shown in the flowchart of the search and selection procedure for eligible studies in the review (Figure 2).

In its November 2024 edition, the JBI Manual for Evidence Synthesis underscores the importance of assessing the quality of evidence, even in scoping reviews. It is recognized that, although this type of review does not aim to critically evaluate all included studies , a systematic description of potential biases, methodological limitations, and evidence heterogeneity is recommended^28^.

According to the manual, at least the overall level of confidence and the characteristics of the studies (e.g., risk of bias, imprecision, inconsistency) should be classified so that reader understands the degree of robustness of the conclusions drawn in the synthesis. This approach contributes to methodological transparency and enables the identification of knowledge gaps, thereby guiding future reviews with more rigorous or specific methodologies^28^.

### Results

The methodological procedures used in the composition of the research corpus were graphically represented by means of a flowchart, produced based on the PRISMA-ScR strategy guidelines for scope reviews, as shown in Figure 2 below:

*VHL = Virtual Health Library

Initially, by applying filters (full text; languages: Portuguese, English, and Spanish) to the selected databases, 200 articles were identified for this review; their data were imported into the Rayyan platform. The Rayyan software automatically summarizes the years of publication of the imported studies; this revealed that the first publication with these descriptors occurred in 2015, but between 2019 and 2023, publications have increased; in 2019, 49 publications were found; in 2020, there were 51; in 2021 and 2022, there were 34 publications in each year; and in 2023, the number of publications found involving the proposed theme fell to 24. This information was extracted from Rayyan.

The final sample comprised eight studies: seven articles published in scientific journals and one doctoral thesis. Regarding country of origin, seven were conducted in Brazil, and one in Romania; all were published in English. The publication period ranged from 2015 to 2022.

The target populations included different types of disabilities: three studies focused on people with hearing impairments, three on people with visual impairments, one on people with intellectual disabilities, and one on people with cognitive disabilities. In terms of the technologies used, digital and printed educational technologies (such as accessible booklets and online courses), assistive technologies (software, avatars, digital accessibility resources), and innovative technologies (exergames) were identified. The topics ranged from oral health, sexual and reproductive health, and sexual abuse prevention to high blood pressure, breast health, and physical activity.

Regarding authorship, most studies were conducted by nursing researchers, with collaborative participation from other disciplines in specific cases, such as physical education, computing, and education. In general, all studies reported positive results regarding the validity, accessibility, acceptability, and feasibility of the technologies, despite small sample sizes, the absence of a control group, and limited follow-up periods. Figure 3 presents the characteristics of the included studies.

**Figure 3:** Description of the included studies. Campina Grande, PB, Brazil, 2024

Nº	Study (author/year)	Type of publication/Country	Target population/Disability	Technology/ Classification/Theme	Key findings	Professional category of authors
1	Nóbrega, et al. (2021)	Article/Brazil	Young people with intellectual disabilities	Validated educational technology/Educational kit with book, support booklet, dolls, and explanatory video, aimed at preventing sexual violence.	High content validity (CVI*≈0.99); potential for prevention, but not tested in the target audience.	Nursing
2	Oliveira, et al. (2020)	Article/Brazil	People with hearing impairments (users of American Sign Language)	Educational technology/Illustrative panel to facilitate health communication between nurses and users.	Sign language resources promote interaction between nurses and deaf people; evidence of acceptability, but no measure of effectiveness.	Nursing
3	Áfio, et al. (2016)	Article/Brazil	People with hearing impairments	Assistive technology/Online course “Sexual and Reproductive Health Education: condom use”	Automatic analysis indicated technical compliance; lack of user testing limited applicability.	Nursing
4	Carvalho, Pagliuca & Fernandes (2015)	Article/Brazil	Visually impaired people (blind)	Educational technology/Online course on breast health.	Initial positive validation of content and accessibility; small sample size.	Nursing
5	Carvalho, et al. (2018)	Article/Brazil	Visually impaired people	Educational technology/Online course on high blood pressure.	Course accessible and considered satisfactory; no clinical impact assessment.	Nursing
6	Marques (2017)	Thesis/Brazil	Visually impaired people (blind)	Educational technology/Virtual primer for the prevention of sexual violence,	Robust methodological validation; positive acceptability; small pilot sample.	Nursing
7	Vaghetti, et al. (2022)	Expanded abstract in proceedings/Brazil	Adults with cognitive disabilities	Innovative technology/Exergames*/*Body movements required by the gameplay promote physical activity.	Exergames promoted socialization and autonomy; feasibility confirmed; absence of control group.	Physical Education and Nursing (collaboration)
8	Chiriac, et al. (2021)	Article/Romania	People with hearing impairments (deaf)	Assistive technology/Avatars and videos for oral health.	Simpler and cheaper videos; more complex avatars; both favored communication.	Multidisciplinary team (Education, Health, and Computing)

*CVI = Content Validity Index

The set analyzed consists predominantly of methodological studies on the development and validation of educational and assistive technologies, experience reports, and exploratory intervention studies, focusing on different types of disabilities (intellectual, visual, auditory, and cognitive). Most studies were conducted in Brazil, except for one in Romania, reflecting a concentration of production in Latin American contexts.

The main types of technologies identified were digital and printed educational technologies: virtual primers and accessible online courses developed for blind people, addressing topics such as sexual health, violence prevention, and high blood pressure. Assistive technologies: accessibility software (screen readers, automatic accessibility assessment), and adaptations for communication in Brazilian Sign Language. Innovative technologies, such as exergames and avatars, are used both to promote physical activity among people with cognitive disabilities and to facilitate oral health communication for deaf people.

Regarding the main findings, the studies indicated that the validated technologies showed high agreement among experts in terms of content and appearance, as in a study^11^ in which the technology on sexual violence prevention for young people with intellectual disabilities obtained a content validity index close to 1.0. Accessible educational resources for blind people, such as virtual primers and online courses, were considered feasible, understandable, and adequate, despite having been tested on small samples and, in general, lacking longitudinal impact assessments.

The use of exergames demonstrated pedagogical feasibility and potential to promote socialization, autonomy, and motor skills, despite the absence of control groups and the heterogeneity of the games employed. The use of avatars and videos in Brazilian Sign Language indicated benefits in health communication for deaf people, but the studies were limited to technical analyses or pilot studies with few participants.

About the assessment of evidence quality, in accordance with the guidelines of the JBI Manual for Evidence Synthesis^28^, a systematic description of potential biases, methodological limitations, and heterogeneity was conducted. In general, the studies had small samples, often selected for convenience, which creates a risk of selection bias and limits generalizability. Many studies have focused solely on validating content and appearance with experts, without testing effectiveness in user populations, thereby limiting the strength of evidence for assessing health impacts.

The heterogeneity across technologies (booklets, courses, software, exergames, avatars) and outcomes (content validity, acceptability, technical compliance, feasibility) precludes direct comparisons or meta-analyses, indicating the need for a narrative synthesis. The overall quality of the evidence was classified as moderate for validity and acceptability, and low for clinical efficacy or behavior change, because none of the studies employed robust designs, such as randomized clinical trials or longitudinal follow-up.

These findings demonstrate that, although existing research is limited in methodological rigor, the studies provide relevant insights into health education practice and the development of inclusive health technologies, particularly regarding the feasibility, accessibility, and cultural appropriateness of resources. The chart constructed (Figure 4), in accordance with the JBI Manual, details the potential biases, methodological limitations, and quality ratings assigned to each included study, serving as a complementary reference to the narrative synthesis.

**Figure 4:** Description of the included studies. Campina Grande, PB, Brazil, 2024

Nº	Type of study	Potential biases	Methodological limitations	Heterogeneity of evidence	Quality of evidence - with justification
1	Methodological study, content/appearance validation by judges	Selection bias: experts by convenience; Disciplinary evaluator bias.	No validation with end users; focus on experts.	Strong evidence only in content validity.	Moderate (content validity), Low (effectiveness). Justification: CVI*=0.99 indicates excellent agreement, but testing was not conducted in young people with ID^†^.
2	Report on experience in Continuing Education	The author reports bias; convenience selection of nurses.	No standardized measures or comparison group.	Evidence of acceptability; does not measure effectiveness.	Low. Justification: shows barriers and the importance of *Libras*, but lacks methodological rigor.
3	Technical conformity assessment	Measurement bias due to automatic analysis; no users.	Not tested with deaf people on a large scale.	Homogeneous technical criteria; not very comparable to clinical studies.	Moderate (compliance), Low (effectiveness). Justification: practical for technical standards, but does not guarantee usability.
4	Methodological study/initial validation	Tiny sample; adjustments made as suggested.	No assessment of impact on behavior or knowledge.	High thematic heterogeneity.	Moderate (initial validity), Low (effectiveness). Justification: robust initial validation, but small scale.
5	Methodological study (Falkembach model)	Project bias (evaluated by developers); small sample size.	Does not evaluate clinical outcomes (e.g., blood pressure control).	High heterogeneity among courses/topics.	Moderate (accessibility), Low (clinical impact). Justification: structured, but ineffective in terms of health.
6	Methodological study, review, FG^‡^, validation, and pilot study (n=22)	Small pilot sample; social response bias.	No longitudinal follow-up; absence of behavioral outcomes.	Consistent evidence of acceptability; few comparable studies.	Moderate (validity/acceptability), Low (impact). Justification: robust, but limited by sample size.
7	Exploratory intervention with 26 participants	Small sample size; observer bias.	No control group; multiple consoles/games.	High heterogeneity between games and outcomes.	Low-Moderate. Justification: shows feasibility but not sustained effectiveness.
8	Comparative technical study	Implementation bias (difference in cost/time); few participants.	Initial results; no robust learning measures.	Specific technical evidence.	Low (effectiveness), Moderate (technical evaluation). Justification: proper for technical decision-making but does not show educational impact.

*CVI = Content Validity Index; ^†^ID = Intellectual Disability; ^‡^FG = Focus Group

## Discussion

This scoping review identified eight studies, primarily methodological, focused on the development and validation of educational and assistive technologies. This predominance reflects the field’s still-emerging nature, in which scientific production focuses on proposing and testing innovative resources rather than on impact studies or systematic reviews. To ensure terminological clarity, it is essential to distinguish between educational technologies (ET), associated with teaching-learning processes, and assistive technologies (AT), aimed at promoting accessibility, autonomy, and inclusion for people with disabilities^5^ ^-^ ^6^. This differentiation guided the analysis of the findings and avoided term overlap.

The data were organized according to the types of technologies identified (educational, assistive, innovative digital), their contexts of application (oral health, sexual and reproductive health, disease prevention, physical activity, and health communication), and the population groups served (people with hearing, visual, intellectual, and cognitive disabilities). This systematization enabled us to understand the diversity of approaches and the convergence of results around common aspects, such as the validity, feasibility, accessibility, and acceptability of technologies.

In the field of sexual and reproductive health, one study stands out for validating an educational technology aimed at preventing sexual violence among young people with intellectual disabilities^11^, bringing to light a topic that is often silenced. Complementing this, another author developed a virtual booklet for people with visual impairments, also focusing on the prevention of sexual violence^29^, reinforcing the potential of educational technologies to address sensitive and neglected issues.

Health communication was another central theme, with emphasis on authors who developed an ET based on *Libras* (Brazilian Sign Language) to facilitate interaction between nurses and deaf people^10^, revealing that language barriers remain obstacles to full inclusion in health services. In the same field, other authors applied the Automatic System for Evaluating Software Accessibility (ASES) tool to digital content, identifying technical compliance but emphasizing the importance of also evaluating the experience of deaf users^30^.

The use of validated instructional *design* models has proven to be strategic for developing accessible technologies. Examples include the online courses on breast health^31^ and high blood pressure^32^, both developed at the Federal University of Ceará. These studies used the Falkembach model and demonstrated that consistent methodologies can ensure quality, accessibility, and safety in the development of digital resources for blind people.

In the area of oral health, an international contribution stands out. A study conducted in Romania compared the use of avatars and videos in applications for the deaf, showing that both resources facilitated communication in oral health, albeit with differences in cost and complexity^33^.

Finally, the use of exergames in physical activity programs for people with cognitive disabilities^34^ has demonstrated benefits related to socialization, motor skill acquisition, and autonomy, and has proven to be a low-cost and viable alternative for promoting health in this population. 

Overall, the findings demonstrate that both ET and AT emerge as valuable resources for promoting inclusive health, contributing to health education, disease prevention, rehabilitation, and social inclusion. The synthesis shows that different areas (sexual and reproductive health, oral health, health communication, and physical activity) can benefit from technologies when they are developed and applied inclusively.

Nevertheless, most studies have focused on the initial stages of development and validation, without extending to analyses of effectiveness in real-world or large-scale contexts. It is essential to emphasize the need for future studies to expand their sample sizes, include control groups, and evaluate the impact of technologies on clinical and social outcomes. Finally, it should be noted that the COVID-19 pandemic, while posing health challenges, has also driven the adoption of inclusive digital tools, paving the way for new studies and innovative practices, but without eliminating access gaps and the need for public policies that ensure sustainability and equity.

## Conclusion

The results revealed that digital and print educational technologies, assistive technologies, and innovative technologies encompass different formats (accessible online courses, digital primers, resources in Brazilian Sign Language, apps, avatars, and *exergames*) and were aimed at various audiences, including people with hearing, visual, intellectual, and cognitive disabilities. Overall, the studies indicated that the technologies developed are well accepted, have valid content, are accessible and viable, and contribute to empowerment, inclusion, and the strengthening of professional health practice, particularly in nursing.

Despite the diversity of proposals, most studies focused on the development and validation stages, without progressing to large-scale effectiveness analyses. In addition, methodological studies and exploration interventions predominated. This characteristic points to both the relevance of the expanding field and the need for more robust investigations, including larger samples, longitudinal follow-up, and controlled comparisons, to strengthen the quality of the evidence.

It is therefore concluded that this study achieved its objective, demonstrating that educational technologies in health for people with disabilities are relevant tools for promoting inclusion and autonomy. However, the review also highlights significant gaps, including limitations in the databases consulted, the geographic concentration of scientific production, and the scarcity of studies that evaluate the concrete impacts of these technologies. It is recommended that future research expand search strategies, diversify research contexts, and advance effective analyses to consolidate the contributions of educational technologies to inclusive health.

## Data Availability

All data generated or analysed during this study are included in this published article.
