# Supplementary material for: Health education technologies for people with disabilities: scope review
Source: Rev Lat Am Enfermagem. 2026 Jul 20;34:e4861. doi: 10.1590/1518-8345.8054.4861 (PMC13387771; doi:10.1590/1518-8345.8054.4861)
Supplement: Translation

# Translation: Tecnologias educacionais em saúde para pessoas com deficiência: revisão de escopo

**DOI:** 10.1590/1518-8345.8054.4862

**Authors:** Danielle Figueiredo Patrício, Alexsandro Silva Coura, Maria do Socorro Alécio Barbosa, Tereza Natália Bezerra de Lima, Morgana Cristina Leôncio de Lima, Kelly Cristina do Nascimento

**Affiliations:** 1 Universidade Estadual da Paraíba, Departamento de Enfermagem, Campina Grande, PB, Brasil.; 2 Scholarship holder at the Fundação de Apoio à Pesquisa do Estado da Paraíba (FAPESQ), Brasil.; 3 Universidade de Pernambuco, Departamento de Enfermagem, Recife, PE, Brasil.; 4 Universidade Federal da Paraíba, Departamento de Enfermagem, João Pessoa, PB, Brasil.

**Keywords:** Tecnologia Educacional, Tecnologia Assistiva, Educação em Saúde, Pessoa com Deficiência, Enfermagem, Revisão.

## Abstract

**Objetivo:**

mapear as tecnologias educacionais desenvolvidas e aplicadas na educação em saúde para pessoas com deficiência.

**Método:**

revisão de escopo baseada nas recomendações do JBI, a partir dos critérios de inclusão: abordar a temática em estudo; texto disponível na íntegra e ser redigido ou traduzido em português, inglês e/ou espanhol. Foram consultadas quatro fontes de informação e literatura cinzenta, no mês de janeiro de 2024.

**Resultados:**

revisão abrangeu oito estudos que abordaram experiências com o planejamento, a construção, a avaliação e a validação de tecnologias voltadas para o processo de educação em saúde de pessoas com deficiência e dos profissionais envolvidos em seus cuidados.

**Conclusão:**

as tecnologias educacionais em saúde às pessoas com deficiência constituem ferramentas para a promoção da inclusão e da autonomia. Predomínio de estudos metodológicos de desenvolvimento e validação, o que mostra lacunas na análise de efetividade e a necessidade de pesquisas mais robustas e diversificadas.

## Destaques

**(1)** As tecnologias educacionais são ferramentas relevantes para pessoas com deficiência. **(2)** Contribuem para a promoção da inclusão e da autonomia da pessoa com deficiência. **(3)** Tem boa aceitação, acessibilidade e potencial de inclusão, especialmente na prática de enfermagem. **(4)** Predomínio de estudos metodológicos e necessidade de pesquisas de efetividade.

## Introdução

As pessoas com deficiência são definidas pela Organização Mundial da Saúde (OMS) como aquelas que apresentam impedimentos físicos, mentais, intelectuais ou sensoriais de longo prazo que, em interação com barreiras sociais, atitudinais e ambientais, podem restringir sua participação plena e efetiva na sociedade em igualdade de condições com as demais pessoas^1^. Estima-se que mais de um bilhão de pessoas no mundo convivam com algum tipo de deficiência, número que pode atingir dois bilhões até 2050 em decorrência do envelhecimento populacional e da maior prevalência de doenças crônicas incapacitantes. No Brasil, dados do Censo de 2022 apontam que 18,6 milhões de pessoas com dois anos ou mais apresentam algum tipo de deficiência, representando 8,9% da população brasileira nessa faixa etária^1^ ^-^ ^2^.

O reconhecimento da deficiência como questão de direitos humanos foi consolidado a partir da Declaração Universal dos Direitos Humanos de 1948 e reafirmado na Convenção sobre os Direitos da Pessoa com Deficiência^3^. Tais documentos estabelecem princípios universais de dignidade, equidade e participação social, exigindo que os Estados garantam políticas públicas voltadas à acessibilidade, inclusão e promoção da autonomia. Essa mudança de paradigma desloca a compreensão da deficiência de um modelo biomédico para uma abordagem social, centrada na eliminação de barreiras e na valorização das capacidades dos indivíduos^4^ ^-^ ^5^.

Nesse contexto, destacam-se as Tecnologias Assistivas (TA), entendidas como recursos, estratégias, produtos e serviços que visam ampliar as habilidades funcionais e a participação social das pessoas com deficiência^6^ ^-^ ^7^. Incluem desde órteses e próteses até *softwares* de leitura de tela, sistemas de comunicação alternativa e adaptações arquitetônicas, configurando-se como ferramentas fundamentais para a autonomia e a inclusão. Em paralelo, as Tecnologias Educacionais (TE), sejam impressas, digitais ou híbridas, têm se mostrado centrais no processo de ensino-aprendizagem em saúde, ao possibilitar a mediação de conhecimentos e a construção de práticas mais acessíveis e participativas^8^ ^-^ ^9^.

A integração de TA e TE responde a desafios práticos enfrentados por pessoas com diferentes tipos de deficiência. Pessoas surdas, por exemplo, enfrentam barreiras comunicacionais nos serviços de saúde, que podem ser minimizadas por aplicativos em Libras e materiais bilíngues^10^. Já indivíduos com deficiência intelectual podem se beneficiar de jogos educativos digitais e recursos visuais simplificados no processo de educação em saúde^11^. Do mesmo modo, *exergames* têm sido aplicados na reabilitação de pessoas com deficiência física ou cognitiva, promovendo ganhos funcionais e sociais por meio de atividades lúdicas^12^. O envelhecimento populacional e o aumento de doenças crônicas também ampliam o contingente de pessoas que adquirem deficiências ao longo da vida, tornando premente a necessidade de ações integrais de cuidado e reabilitação^13^. Para além da dimensão clínica, o processo de educação em saúde constitui ferramenta estratégica, pois promove não apenas a transmissão de informações, mas também a formação de consciência crítica, favorecendo a autonomia, o protagonismo e a transformação social^14^ ^-^ ^15^.

Nesse ínterim, a articulação entre tecnologias assistivas e educação em saúde constitui eixo fundamental para ampliar a autonomia e inclusão das pessoas com deficiência. As tecnologias assistivas, ao oferecer recursos que favorecem a funcionalidade e participação social, tornam-se instrumentos estratégicos quando associadas a processos educativos, potencializando o acesso à informação e promoção da saúde. Nesse cenário, a enfermagem assume papel central por sua proximidade contínua com os usuários, mediando o uso dessas tecnologias, traduzindo informações em linguagem acessível e estimulando o protagonismo dos sujeitos no cuidado de si. Essa articulação amplia não apenas os resultados clínicos e reabilitadores, mas também fortalece as dimensões sociais e cidadãs do cuidado^5^ ^-^ ^6^ ^,^ ^11^.

Nesse contexto, as TE despontam como recursos essenciais no ensino-aprendizagem em saúde. Elas podem assumir diferentes tipologias: tecnologias assistivas (recursos que favorecem funcionalidade e autonomia, como próteses, softwares de leitura e adaptações físicas); tecnologias educacionais tradicionais (cartilhas, guias, manuais, jogos impressos); e tecnologias digitais inovadoras, como *exergames* e aplicativos interativos, que integram ludicidade, atividade física e aprendizado^7^ ^-^ ^8^ ^,^ ^16^. Todas elas compartilham o potencial de transformar o processo educativo em um espaço dialógico e participativo, fortalecendo a cidadania e inclusão social^17^ ^-^ ^20^.

As práticas educativas em saúde, quando direcionadas às pessoas com deficiência, devem articular cuidado direto e indireto, integrando dimensões físicas, subjetivas e sociais. Profissionais de enfermagem, por sua atuação próxima e contínua, assumem papel central nesse processo, tanto na reabilitação quanto na promoção da inclusão e do empoderamento, indo além do modelo curativo e reconhecendo a pessoa com deficiência como sujeito ativo do cuidado^21^ ^-^ ^22^.

Diante desse cenário, este estudo tem como objetivo mapear as tecnologias educacionais desenvolvidas e aplicadas na educação em saúde para pessoas com deficiência.

## Método

Trata-se de uma revisão de escopo, que possibilitou o mapeamento de evidências ao explorar a amplitude e a extensão da literatura, com o objetivo de identificar lacunas e sugerir estudos futuros^23^. A revisão de escopo constitui um tipo de pesquisa que fornece uma visão geral sobre a dimensão e a quantidade de estudos disponíveis, tendo como finalidade identificar a natureza e a extensão da produção científica^24^.

Esta pesquisa foi baseada no guia para revisão de escopo do JBI, que destaca as etapas do processo de construção: 1) Definir e alinhar os objetivos e a pergunta de pesquisa; 2) Desenvolver e alinhar os critérios de inclusão com os objetivos e a pergunta de pesquisa; 3) Descrever a abordagem planejada para identificar as evidências; 4) Pesquisar as evidências; 5) Selecionar as evidências; 6) Extrair os dados; 7) Apresentar os resultados em gráficos e tabelas; 8) Resumir os achados em relação aos objetivos e à pergunta de pesquisa^23^. O protocolo deste estudo está publicado na plataforma *Open Science Framework*: https://osf.io/a547g/.

### Identificação da pergunta de pesquisa

Para elaboração da questão norteadora e delineamento da busca, foi utilizada a estratégia PICo. Essa estratégia contempla os seguintes elementos sintetizados no acrônimo: P= Problema (Educação em Saúde); I = Interesse (Tecnologia Educacional) e Co = Contexto (Pessoas com Deficiência). Para tanto, a questão norteadora desta pesquisa é: quais são as tecnologias educativas utilizadas para promover educação em saúde nas pessoas com deficiência?

### Estratégia de pesquisa

Dentre os portais virtuais disponíveis para coleta de dados, foram selecionadas as bases *Medical Literature Analysis and Retrieval System Online* (MEDLINE), *Excerpta Medica database* (Embase), Biblioteca Virtual em Saúde (BVS), Literatura Latino-Americana e do Caribe em Ciências da Saúde (LILACS) e Google *Scholar* (para literatura cinzenta), por serem amplamente reconhecidas no campo da saúde. O objetivo foi analisar a produção científica acerca da temática proposta em contextos nacional e internacional.

A busca avançada dos artigos foi realizada em janeiro de 2024, simultaneamente nas cinco bases. A seleção e a avaliação dos estudos foram conduzidas de maneira independente e cegada por dois revisores, e eventuais discordâncias foram resolvidas em consenso com um terceiro revisor^25^. Para selecionar os estudos iniciais e excluir duplicados, os revisores procederam à leitura dos títulos e resumos utilizando o gerenciador de referências online Rayyan. A seleção elaborada por cada revisor foi sistematicamente ordenada com apoio do software e, posteriormente, comparada entre si para validação da amostra final^26^.

As palavras-chave foram selecionadas a partir dos Descritores em Ciências da Saúde (DeCS), sendo elas: “Tecnologia educacional”, “Educational Technology”, “Pessoa com deficiência”, “Disabled Persons”, “Educação em saúde” e “Health Education”, utilizando-se os operadores booleanos OR e AND. Foram combinadas de diferentes formas, como: *Tecnologia educacional OR Educação em Saúde AND Pessoa com deficiência*; *Tecnologia educacional AND Educação em Saúde AND Pessoa com deficiência*, em português e inglês. Os resultados estão dispostos na Figura 1:

**Figura 1:** Tabela de descrição dos operadores booleanos para busca por base. Campina Grande, PB, Brasil, 2024

Mecanismo de busca	Termos de pesquisa	Citações recuperadas
MEDLINE	*(“Educational Technology”\[MeSH Terms] OR “educational technology”\[tiab] OR “tecnologia educacional”\[tiab]) AND (“Health Education”\[MeSH Terms] OR “health education”\[tiab] OR “educação em saúde”\[tiab]) AND (“Disabled Persons”\[MeSH Terms] OR “people with disabilities”\[tiab] OR “pessoa com deficiência”\[tiab])*	78
Embase	*(‘educational technology’/exp OR ‘educational technology’\:ti,ab OR ‘tecnologia educacional’\:ti,ab) AND (‘health education’/exp OR ‘health education’\:ti,ab OR ‘educação em saúde’\:ti,ab) AND (‘disabled person’/exp OR ‘people with disabilities’\:ti,ab OR ‘pessoa com deficiência’\:ti,ab)*	57
BVS	*(“Tecnologia Educacional” OR “tecnologia educacional” OR “Educational Technology”) AND (“Educação em Saúde” OR “Health Education”) AND (“Pessoas com Deficiência” OR “pessoa com deficiência” OR “People with Disabilities”)*	23
LILACS	*(“Tecnologia Educacional” OR “Educational Technology”) AND (“Educação em Saúde” OR “Health Education”) AND (“Pessoas com Deficiência” OR “People with Disabilities”)*	29
Literatura cinzenta	*(“tecnologia educacional” OR “educational technology”) AND (“educação em saúde” OR “health education”) AND (“pessoa com deficiência” OR “people with disabilities”)*	13
**Total**		200

### Critérios de elegibilidade

A seleção dos estudos analisados ocorreu a partir dos critérios de inclusão: abordar a temática em estudo; texto disponível na íntegra e ser redigido ou traduzido em português, inglês e/ou espanhol. Os critérios de exclusão foram: textos incompletos ou com acesso indisponível; repetição de um mesmo artigo em mais de uma base de dados; falta de relação com o objeto de estudo. Não foi considerado recorte no tempo de pesquisa com intuito de resgatar o maior número de estudos possíveis.

### Tratamento e análise dos dados

Utilizaram-se as diretrizes da estratégia PRISMA-ScR para revisões de escopo^27^, para representar graficamente o procedimento de triagem dos estudos, como mostra o fluxograma do procedimento de busca e seleção dos estudos elegíveis na revisão (Figura 2).

Em sua edição de novembro de 2024, o JBI *Manual for Evidence Synthesis* reforça a importância de abordar a qualidade da evidência mesmo em revisões de escopo. Reconhece-se que, embora esse tipo de revisão não tenha como objetivo avaliar criticamente todos os estudos incluídos, torna-se recomendável uma descrição sistemática de vieses potenciais, limitações metodológicas e heterogeneidade das evidências^28^.

Segundo o manual, deve-se ao menos classificar o nível de confiança global e as características dos estudos (por exemplo: risco de viés, imprecisão, inconsistência), para que o leitor compreenda o grau de robustez das conclusões extraídas na síntese. Esse enfoque contribui para a transparência metodológica e permite identificar lacunas de conhecimento, orientando futuras revisões com metodologias mais rigorosas ou específicas^28^.

## Resultados

Os procedimentos metodológicos utilizados na composição do *corpus* de pesquisa foram graficamente representados por meio de um fluxograma, produzido a partir das diretrizes da estratégia PRISMA-ScR para revisões de escopo conforme Figura 2 a seguir:

**Figura 2:** Fluxograma PRISMA-ScR utilizado para identificação e seleção dos estudos. Campina Grande, PB, Brasil, 2024

Inicialmente, com aplicação de filtros (texto na íntegra; língua: português, inglês e espanhol) nas bases de dados selecionadas, foram identificados 200 artigos por meio das pesquisas para essa revisão; os dados desses artigos foram importados na plataforma Rayyan. O *software* do Rayyan faz um resumo automático acerca dos anos de publicações dos estudos importados; com isso, surgiu a informação de que a primeira publicação com esses descritores ocorreu no ano de 2015, porém, entre 2019 e 2023, as publicações aumentaram; de 2019, houve 49 publicações encontradas, em 2020 foram 51, em 2021 e 2022 foram 34 publicações em cada ano e, em 2023, o número de publicações encontradas envolvendo o tema proposto caiu para 24. Essas informações foram extraídas do Rayyan.

A amostra final foi composta por oito estudos, sendo sete artigos publicados em periódicos científicos e uma tese de doutorado. Quanto ao país de origem, sete foram realizados no Brasil e um na Romênia, publicado em inglês. O período de publicação variou entre 2015 e 2022.

As populações-alvo contemplaram diferentes tipos de deficiência: três estudos voltados a pessoas com deficiência auditiva, três a pessoas com deficiência visual, um a pessoas com deficiência intelectual e um a pessoas com deficiência cognitiva. Em relação às tecnologias utilizadas, identificaram-se tecnologias educacionais digitais e impressas (como cartilhas e cursos online acessíveis), tecnologias assistivas (*softwares*, avatares, recursos de acessibilidade digital) e tecnologias inovadoras (*exergames*). As temáticas abordaram desde saúde bucal, saúde sexual e reprodutiva e prevenção do abuso sexual até hipertensão arterial, saúde mamária e atividade física.

No que se refere à autoria, a maioria das produções foi conduzida por pesquisadores da enfermagem, com participação colaborativa de outras áreas em casos pontuais, como educação física, computação e educação. De forma geral, todos os estudos reportaram resultados positivos em termos de validade, acessibilidade, aceitabilidade e viabilidade das tecnologias, ainda que limitados por amostras pequenas, ausência de grupo controle e curta duração de acompanhamento. A Figura 3 a seguir mostra a caracterização dos estudos incluídos.

**Figura 3:** Descrição dos estudos incluídos. Campina Grande, PB, Brasil, 2024

Nº	Estudo (autor/ano)	Tipo de publicação/País	População-alvo/Deficiência	Tecnologia/ Classificação/Tema	Principais achados	Categoria profissional dos autores
1	Nóbrega, et al. (2021)	Artigo/Brasil	Jovens com deficiência intelectual	Tecnologia educacional validada/Kit educativo com livro, livreto de apoio, bonecos e vídeo explicativo, direcionada para a prevenção da violência sexual.	Alta validade de conteúdo (IVC*≈0,99); potencial para prevenção, mas não testado em público-alvo.	Enfermagem
2	Oliveira, et al. (2020)	Artigo/Brasil	Pessoas com deficiência auditiva (usuários de Libras)	Tecnologia educacional/Painel ilustrativo para facilitar a comunicação em saúde entre enfermeiros e usuários.	Recurso em Libras favorece interação enfermeiro-pessoa surda; evidência de aceitabilidade, sem medir eficácia.	Enfermagem
3	Áfio, et al. (2016)	Artigo/Brasil	Pessoas com deficiência auditiva	Tecnologia assistiva/Curso online “Educação em Saúde Sexual e Reprodutiva: uso dos preservativos”	Análise automática indicou conformidade técnica; ausência de testes com usuários limitou a aplicabilidade.	Enfermagem
4	Carvalho, Pagliuca & Fernandes (2015)	Artigo/Brasil	Pessoas com deficiência visual (cegas)	Tecnologia educacional/ Curso online em saúde mamária.	Validação inicial positiva de conteúdo e acessibilidade; amostra pequena.	Enfermagem
5	Carvalho, et al. (2018)	Artigo/Brasil	Pessoas com deficiência visual	Tecnologia educacional/ Curso online sobre hipertensão arterial.	Curso acessível e considerado satisfatório; sem avaliação de impacto clínico.	Enfermagem
6	Marques (2017)	Tese/Brasil	Pessoas com deficiência visual (cegas)	Tecnologia educacional/Cartilha virtual para prevenção da violência sexual.	Validação metodológica robusta; aceitabilidade positiva; amostra-piloto pequena.	Enfermagem
7	Vaghetti, et al. (2022)	Resumo expandido em anais/Brasil	Adultos com deficiência cognitiva	Tecnologia digital inovadora/*Exergames*/Movimento corporais exigidos pelos *gameplay* utilizados promovendo atividade física.	*Exergames* **promoveram socialização e autonomia; viabilidade confirmada; ausência de grupo controle.**	Educação Física e Enfermagem (colaboração)
8	Chiriac, et al. (2021)	Artigo/Romênia	Pessoas com deficiência auditiva (surdas)	Tecnologia assistiva/Avatares e vídeos para saúde bucal.	Vídeos mais simples e baratos; avatares mais complexos; ambos favoreceram a comunicação.	Equipe multidisciplinar (Educação, Saúde e Computação)

*IVC = Índice de Validade de Conteúdo

O conjunto analisado é composto predominantemente por estudos metodológicos de desenvolvimento e validação de tecnologias educacionais e assistivas, relatos de experiência e estudos exploratórios de intervenção, com foco em diferentes tipos de deficiência (intelectual, visual, auditiva e cognitiva). A maioria dos estudos foi desenvolvida no Brasil, com exceção de um estudo realizado na Romênia, refletindo uma produção concentrada em contextos latino-americanos.

Os principais tipos de tecnologias identificados foram: tecnologias educacionais digitais e impressas: cartilhas virtuais e cursos online acessíveis, desenvolvidos para pessoas cegas, e abordando temas como saúde sexual, prevenção de violência e hipertensão arterial. Tecnologias assistivas: *softwares* de acessibilidade (leitores de tela, avaliação automática de acessibilidade) e adaptações para comunicação em Libras. Tecnologias inovadoras: *exergames* e avatares, utilizados tanto para promover atividade física em pessoas com deficiência cognitiva quanto para facilitar a comunicação em saúde bucal de pessoas surdas. Em relação aos principais achados, os estudos apontaram que as tecnologias validadas apresentaram alta concordância entre especialistas quanto ao conteúdo e aparência, como em um estudo^11^ ^)^ em que a tecnologia sobre prevenção da violência sexual para jovens com deficiência intelectual obteve índice de validade de conteúdo próximo a 1,0. Recursos educacionais acessíveis para pessoas cegas, como cartilhas virtuais e cursos online, foram considerados viáveis, compreensíveis e adequados, embora testados em amostras pequenas e, em geral, sem avaliação longitudinal de impacto.

A utilização de *exergames* mostrou viabilidade pedagógica e potencial para promover socialização, autonomia e habilidades motoras, apesar da ausência de grupos controle e da heterogeneidade dos tipos de jogos aplicados. O uso de avatares e vídeos em Libras indicou benefícios na comunicação em saúde para pessoas surdas, mas os estudos se limitaram a análises técnicas ou a pilotos com poucos participantes.

No que se refere à avaliação da qualidade das evidências, em conformidade com as orientações do JBI *Manual for Evidence Synthesis* ^28^, foi realizada uma descrição sistemática dos vieses potenciais, limitações metodológicas e heterogeneidade. De modo geral: os estudos apresentaram amostras reduzidas, frequentemente selecionadas por conveniência, o que gera risco de viés de seleção e limita a generalização. Muitos trabalhos concentraram-se apenas em validar conteúdo e aparência com especialistas, sem testar efetividade em populações usuárias, restringindo a força da evidência para avaliar o impacto em saúde.

A heterogeneidade entre as tecnologias (cartilhas, cursos, *softwares*, *exergames*, avatares) e entre os desfechos (validade de conteúdo, aceitabilidade, conformidade técnica, viabilidade) impossibilita comparações diretas ou metanálises, indicando a necessidade de uma síntese narrativa. A qualidade geral da evidência foi classificada como moderada para validade e aceitabilidade e baixa para eficácia clínica ou mudança de comportamento, já que nenhum dos estudos empregou desenhos robustos como ensaios clínicos randomizados ou acompanhamentos longitudinais.

Esses achados mostram que, embora a produção existente seja limitada em termos de rigor metodológico, os estudos fornecem subsídios relevantes para a prática da educação em saúde e para o desenvolvimento de tecnologias inclusivas em saúde, principalmente no que tange à viabilidade, acessibilidade e adequação cultural dos recursos. O quadro construído (Figura 4), em conformidade com o Manual JBI, apresenta detalhadamente os vieses potenciais, limitações metodológicas e a qualidade atribuída a cada estudo incluído, servindo como referência complementar à síntese narrativa.

**Figura 4:** Descrição dos estudos incluídos. Campina Grande, PB, Brasil, 2024

Nº	Tipo de estudo	Viés potencial	Limitações metodológica	Heterogeneidade das evidências	Qualidade da evidência - com justificativa
1	Estudo metodológico, validação de conteúdo/aparência por juízes	Viés de seleção: especialistas por conveniência; Viés de avaliador disciplinar.	Não houve validação com usuários finais; foco em especialistas.	Evidência forte apenas em validade de conteúdo.	Moderada (validade de conteúdo), Baixa (eficácia). Justificativa: IVC*=0,99 indica excelente concordância, mas sem testagem em jovens com DI^†^.
2	Relato de experiência em Educação Permanente	Viés de relato dos autores; seleção por conveniência de enfermeiros.	Sem medidas padronizadas ou grupo de comparação.	Evidência de aceitabilidade; não mede eficácia.	Baixa. Justificativa: mostra barreiras e importância da Libras, mas sem rigor metodológico.
3	Avaliação técnica de conformidade	Viés de mensuração por análise automática; sem usuários.	Não testado com surdos em larga escala.	Homogêneo em critérios técnicos; pouco comparável a estudos clínicos.	Moderada (conformidade), Baixa (eficácia). Justificativa: útil para padrões técnicos, mas não garante usabilidade.
4	Estudo metodológico/validação inicial	Amostra muito pequena; ajustes feitos conforme sugestões.	Sem avaliação de impacto em comportamento ou conhecimento.	Alta heterogeneidade temática.	Moderada (validade inicial), Baixa (eficácia). Justificativa: validação inicial robusta, mas pequena escala.
5	Estudo metodológico (modelo Falkembach)	Viés de projeto (desenvolvedoras avaliaram); amostra pequena.	Não avalia desfechos clínicos (por ex. controle da pressão arterial).	Alta heterogeneidade entre cursos/temas.	Moderada (acessibilidade), Baixa (impacto clínico). Justificativa: estruturado, mas sem efetividade em saúde.
6	Estudo metodológico, revisão, GF^‡^, validação e piloto (n=22)	Amostra piloto pequena; viés de resposta social.	Sem seguimento longitudinal; ausência de desfechos comportamentais.	Evidência consistente de aceitabilidade; poucos estudos comparáveis.	Moderada (validade/aceitabilidade), baixa (impacto). Justificativa: robusto, mas limitado por tamanho da amostra.
7	Intervenção exploratória com 26 participantes	Amostra pequena; viés de observador.	Sem grupo controle; múltiplos consoles/jogos.	Heterogeneidade elevada entre jogos e desfechos.	Baixa-Moderada. Justificativa: mostra viabilidade, mas não eficácia sustentada.
8	Estudo comparativo técnico	Viés de implementação (diferença de custo/tempo); poucos participantes.	Resultados iniciais; sem medidas robustas de aprendizagem.	Evidência técnica específica.	Baixa (eficácia), Moderada (avaliação técnica). Justificativa: útil para decisão técnica, mas não mostra impacto educativo.

*IVC = Índice de Validade de Conteúdo; ^†^DI = Deficiência Intelectual; ^‡^GF = Grupo Focal

## Discussão

A presente revisão de escopo identificou oito estudos, em sua maioria metodológicos, voltados ao desenvolvimento e validação de tecnologias educacionais e assistivas. Essa predominância reflete a natureza ainda emergente do campo, em que a produção científica se concentra em propor e testar recursos inovadores, mais do que em estudos de impacto ou revisões sistemáticas. Para garantir clareza terminológica, é importante distinguir as tecnologias educacionais (TE), associadas a processos de ensino-aprendizagem, das tecnologias assistivas (TA), voltadas à promoção de acessibilidade, autonomia e inclusão de pessoas com deficiência^5^ ^-^ ^6^. Essa diferenciação orientou a análise dos achados e evita a sobreposição de termos.

Os dados foram organizados de acordo com os tipos de tecnologias identificadas (educacionais, assistivas, digitais inovadoras), seus contextos de aplicação (saúde bucal, saúde sexual e reprodutiva, prevenção de agravos, atividade física e comunicação em saúde) e os grupos populacionais atendidos (pessoas com deficiência auditiva, visual, intelectual e cognitiva). Essa sistematização permitiu compreender a diversidade de abordagens e a convergência dos resultados em torno de aspectos comuns, como validade, viabilidade, acessibilidade e aceitação das tecnologias.

No campo da saúde sexual e reprodutiva, destaca-se o estudo que validou uma tecnologia educacional voltada à prevenção da violência sexual em jovens com deficiência intelectual^11^, trazendo à tona um tema frequentemente silenciado. De forma complementar, outro autor desenvolveu uma cartilha virtual para pessoas com deficiência visual, também com foco na prevenção da violência sexual^29^, reforçando o potencial das tecnologias educacionais para abordar questões sensíveis e negligenciadas.

A comunicação em saúde foi outro eixo central, com destaque aos autores que desenvolveram uma TE baseada na Libras para facilitar a interação entre enfermeiros e pessoas surdas^10^, revelando que barreiras de linguagem ainda são obstáculos à inclusão plena nos serviços de saúde. Nesse mesmo campo, outros autores aplicaram o instrumento *Automatic System for Evaluating Software Accessibility* (ASES) em conteúdos digitais, identificando conformidade técnica, mas ressaltando a importância de avaliar também a experiência real dos usuários surdos^30^.

O uso de modelos validados de *design* instrucional mostrou-se estratégico para a construção de tecnologias acessíveis. Exemplo disso são os cursos online sobre saúde mamária^31^ e sobre hipertensão arterial^32^, ambos desenvolvidos na Universidade Federal do Ceará. Esses estudos utilizaram o modelo de Falkembach e demonstraram que metodologias consistentes podem assegurar qualidade, acessibilidade e segurança na elaboração de recursos digitais para pessoas cegas.

No eixo da saúde bucal, destaca-se uma contribuição internacional. O estudo realizado na Romênia comparou o uso de avatares e vídeos em aplicativos para surdos, mostrando que ambos os recursos favoreceram a comunicação em saúde oral, ainda que com diferenças de custo e complexidade^33^.

Por fim, a utilização de *exergames* em programas de atividade física para pessoas com deficiência cognitiva^34^ revelou benefícios relacionados à socialização, ganho de habilidades motoras e autonomia, mostrando-se uma alternativa de baixo custo e viável para a promoção da saúde nesse público.

De forma geral, os achados mostram que tanto as TE quanto as TA emergem como recursos valiosos para a promoção da saúde inclusiva, contribuindo para a educação em saúde, a prevenção de agravos, a reabilitação e a inclusão social. A síntese realizada evidencia que diferentes áreas (saúde sexual e reprodutiva, saúde bucal, comunicação em saúde e atividade física) podem se beneficiar de tecnologias quando desenvolvidas e aplicadas de forma inclusiva.

Ainda assim, cabe destacar que a maioria dos estudos se concentrou em etapas iniciais de desenvolvimento e validação, sem avançar para análises de efetividade em contextos reais ou em larga escala. Vale reforçar a importância de futuros trabalhos ampliarem amostras, incluírem grupo controle e avaliarem o impacto das tecnologias em desfechos clínicos e sociais. Por fim, ressalta-se que a pandemia de COVID-19, embora tenha imposto desafios sanitários, também impulsionou a adoção de ferramentas digitais inclusivas, abrindo caminho para novos estudos e práticas inovadoras, mas sem eliminar as lacunas de acesso e a necessidade de políticas públicas que assegurem sustentabilidade e equidade.

## Conclusão

Os resultados evidenciam que as tecnologias educacionais digitais e impressas, tecnologias assistivas e tecnologias inovadoras contemplam diferentes formatos (cursos online acessíveis, cartilhas digitais, recursos em Libras, aplicativos, avatares e *exergames*) e foram direcionadas a variados públicos, incluindo pessoas com deficiência auditiva, visual, intelectual e cognitiva. De modo geral, os estudos mostraram que as tecnologias desenvolvidas apresentam boa aceitação, validade de conteúdo, acessibilidade e viabilidade, contribuindo para o empoderamento, a inclusão e o fortalecimento da prática profissional em saúde, especialmente na enfermagem.

Apesar da diversidade de propostas, observou-se que a maioria das pesquisas se concentrou em etapas de desenvolvimento e validação, sem avançar para análises de efetividade em larga escala. Além disso, houve a predominância de estudos metodológicos e de intervenções exploratórias. Essa característica aponta tanto para a relevância do campo em expansão quanto para a necessidade de investigações mais robustas, com amostras maiores, seguimento longitudinal e comparações controladas, a fim de fortalecer a qualidade da evidência.

Considera-se, portanto, que este estudo atingiu seu objetivo, mostrando que as tecnologias educacionais em saúde voltadas às pessoas com deficiência são ferramentas relevantes para a promoção da inclusão e da autonomia. Entretanto, a revisão também evidencia lacunas importantes, como a limitação de bases de dados consultadas, a concentração geográfica da produção científica e a escassez de estudos avaliando impactos concretos dessas tecnologias. Recomenda-se que futuras pesquisas ampliem as estratégias de busca, diversifiquem os contextos de investigação e avancem em análises de efetividade, de modo a consolidar a contribuição das tecnologias educacionais para a saúde inclusiva.

## Data Availability

Todos os dados gerados ou analisados durante este estudo estão incluídos neste artigo publicado.

# Translation: Tecnologías educativas en salud para personas con discapacidad: revisión del alcance

**DOI:** 10.1590/1518-8345.8054.4860

**Authors:** Danielle Figueiredo Patrício, Alexsandro Silva Coura, Maria do Socorro Alécio Barbosa, Tereza Natália Bezerra de Lima, Morgana Cristina Leôncio de Lima, Kelly Cristina do Nascimento

**Affiliations:** 1 Universidade Estadual da Paraíba, Departamento de Enfermagem, Campina Grande, PB, Brasil.; 2 Scholarship holder at the Fundação de Apoio à Pesquisa do Estado da Paraíba (FAPESQ), Brasil.; 3 Universidade de Pernambuco, Departamento de Enfermagem, Recife, PE, Brasil.; 4 Universidade Federal da Paraíba, Departamento de Enfermagem, João Pessoa, PB, Brasil.

**Keywords:** Tecnología Educativa, Tecnología de Asistencia, Educación para la Salud, Persona con Discapacidad, Enfermería, Revisión.

## Abstract

**Objetivo:**

mapear las tecnologías educativas desarrolladas y aplicadas en la educación en salud para personas con discapacidad.

**Método:**

revisión de alcance basada en las recomendaciones de JBI, según los criterios de inclusión: abordar el tema en estudio; texto disponible en su totalidad y ser escrito o traducido al portugués, inglés y/o español. Se consultaron cuatro fuentes de información y literatura gris en enero de 2024.

**Resultados:**

a revisión abarcó ocho estudios que abordaron experiencias de planificación, construcción, evaluación y validación de tecnologías dirigidas al proceso de educación en salud de personas con discapacidad y los profesionales involucrados en su atención.

**Conclusión:**

las tecnologías educativas en salud para personas con discapacidad constituyen herramientas para promover la inclusión y la autonomía. El predominio de estudios de desarrollo y validación metodológica revela lagunas en el análisis de efectividad y la necesidad de una investigación más sólida y diversificada.

## Destacados

**(1)** Las tecnologías educativas son herramientas relevantes para las personas con discapacidad. **(2)** Contribuyen a promover la inclusión y la autonomía de las personas con discapacidad. **(3)** Tienen buena aceptación, accesibilidad y potencial de inclusión, especialmente en la práctica enfermera. **(4)** Predominan los estudios metodológicos y se necesita investigación sobre su eficacia.

## Introducción

Las personas con discapacidad son definidas por la Organización Mundial de la Salud (OMS) como aquellas que tienen discapacidades físicas, mentales, intelectuales o sensoriales a largo plazo que, en interacción con barreras sociales, actitudinales y ambientales, pueden restringir su participación plena y efectiva en la sociedad en igualdad de condiciones con otras personas^1^. Se estima que más de mil millones de personas en el mundo viven con algún tipo de discapacidad, cifra que podría alcanzar los dos mil millones en 2050 debido al envejecimiento de la población y a la mayor prevalencia de enfermedades crónicas incapacitantes. En Brasil, los datos del Censo de 2022 indican que 18,6 millones de personas de dos años o más tienen algún tipo de discapacidad, lo que representa el 8,9% de la población brasileña en este grupo de edad^1^ ^-^ ^2^.

El reconocimiento de la discapacidad como cuestión de derechos humanos se consolidó a partir de la Declaración Universal de los Derechos Humanos de 1948 y se reafirmó en la Convención sobre los Derechos de las Personas con Discapacidad^3^. Estos documentos establecen principios universales de dignidad, equidad y participación social, exigiendo a los Estados que garanticen políticas públicas orientadas a la accesibilidad, la inclusión y la promoción de la autonomía. Este cambio de paradigma traslada la comprensión de la discapacidad de un modelo biomédico a un enfoque social, centrado en la eliminación de barreras y la valoración de las capacidades individuales^4^ ^-^ ^5^.

En este contexto, las Tecnologías Asistivas (TA) destacan, entendidas como recursos, estrategias, productos y servicios que buscan ampliar las habilidades funcionales y la participación social de las personas con discapacidad^6^ ^-^ ^7^. Van desde ortesis y prótesis hasta *software* de lectura de pantalla, sistemas alternativos de comunicación y adaptaciones arquitectónicas, configurándose como herramientas fundamentales para la autonomía y la inclusión. Paralelamente, las Tecnologías Educativas (TE), ya sean impresas, digitales o híbridas, han demostrado ser centrales en el proceso de enseñanza-aprendizaje en salud, al permitir la mediación del conocimiento y la construcción de prácticas más accesibles y participativas^8^ ^-^ ^9^.

La integración de TA y TE responde a los desafíos prácticos que enfrentan las personas con diferentes tipos de discapacidades. Las personas sordas, por ejemplo, enfrentan barreras de comunicación en los servicios de salud, que pueden minimizarse mediante aplicaciones en *Libras* y materiales bilingües^10^. Las personas con discapacidad intelectual, por otro lado, pueden beneficiarse de juegos educativos digitales y recursos visuales simplificados en el proceso de educación para la salud^11^. De manera similar, los ejercicios se han aplicado en la rehabilitación de personas con discapacidades físicas o cognitivas, promoviendo avances funcionales y sociales a través de actividades lúdicas^12^. El envejecimiento de la población y el aumento de enfermedades crónicas también incrementan el número de personas que adquieren discapacidades a lo largo de su vida, haciendo urgente la necesidad de una atención integral y acciones de rehabilitación^13^. Además de la dimensión clínica, el proceso de educación sanitaria es una herramienta estratégica, ya que promueve no solo la transmisión de información, sino también la formación de una conciencia crítica, favoreciendo la autonomía, el protagonismo y la transformación social^14^ ^-^ ^15^.

Mientras tanto, la articulación entre las tecnologías asistenciales y la educación sanitaria es un eje fundamental para ampliar la autonomía e inclusión de las personas con discapacidad. Al ofrecer recursos que favorecen la funcionalidad y la participación social, las tecnologías de asistencia se convierten en instrumentos estratégicos cuando se asocian con procesos educativos, mejorando el acceso a la información y la promoción de la salud. En este escenario, la enfermería asume un papel central debido a su proximidad continua con los usuarios, mediando el uso de estas tecnologías, traduciendo la información en lenguaje accesible y estimulando el protagonismo de los sujetos en el autocuidado. Esta articulación amplía no solo los resultados clínicos y rehabilitadores, sino que también fortalece las dimensiones sociales y ciudadanas de la atención^5^ ^-^ ^6^ ^,^ ^11^.

En este contexto, las Tecnologías Educativas (TE) emergen como recursos esenciales en la enseñanza y el aprendizaje de la salud. Pueden adoptar diferentes tipologías: tecnologías asistenciales (recursos que favorecen la funcionalidad y la autonomía, como prótesis, *software* de lectura y adaptaciones físicas); tecnologías educativas tradicionales (folletos, guías, manuales, juegos impresos); y tecnologías digitales innovadoras, como los *exergames* (videojuegos) y las aplicaciones interactivas, que integran la ludicidez, la actividad física y el aprendizaje^7^ ^-^ ^8^ ^,^ ^16^. Todos ellos comparten el potencial de transformar el proceso educativo en un espacio dialogado y participativo, fortaleciendo la ciudadanía y la inclusión social^17^ ^-^ ^20^.

Las prácticas educativas en salud, cuando se dirigen a personas con discapacidad, deben articular la atención directa e indirecta, integrando las dimensiones física, subjetiva y social. Los profesionales de enfermería, debido a su trabajo cercano y continuo, desempeñan un papel central en este proceso, tanto en la rehabilitación como en la promoción de la inclusión y el empoderamiento, yendo más allá del modelo curativo y reconociendo a la persona con discapacidad como un sujeto activo de atención^21^ ^-^ ^22^. 

En vista de este escenario, este estudio pretende mapear las tecnologías educativas desarrolladas y aplicadas en la educación sanitaria para personas con discapacidad.

## Método

Se trata de una revisión de alcance que permitió mapear la evidencia explorando la amplitud y extensión de la literatura, con el objetivo de identificar lagunas y sugerir futuros estudios^23^. La revisión por alcance es un tipo de investigación que ofrece una visión general del tamaño y la cantidad de estudios disponibles, con el propósito de identificar la naturaleza y el alcance de la producción científica^24^.

Esta investigación se basó en la guía del JBI para la revisión del alcance, que destaca los pasos del proceso de construcción: 1) Definir y alinear los objetivos y la pregunta de investigación; 2) Desarrollar y alinear los criterios de inclusión con los objetivos y la pregunta de investigación; 3) Describir el enfoque planificado para identificar las pruebas; 4) Investigar la evidencia; 5) Seleccionar la evidencia; 6) Extraer los datos; 7) Presentar los resultados en gráficos y tablas; 8) Resumir los hallazgos en relación con los objetivos y la pregunta de investigación^23^. El protocolo de este estudio se publica en el *Open Science Framework*: https://osf.io/a547g/.

### Identificación de la pregunta de la investigación

Para elaborar la pregunta guía y diseñar la búsqueda, se utilizó la estrategia PICo. Esta estrategia incluye los siguientes elementos resumidos en el acrónimo: P = Problema (Educación para la Salud); I = Interés (Tecnología Educativa) y Co = Contexto (Personas con Discapacidad). Por lo tanto, la pregunta guía de esta investigación es: ¿cuáles son las tecnologías educativas utilizadas para promover la educación sanitaria en personas con discapacidad?

### Estrategia de investigación

Entre los portales virtuales disponibles para la recopilación de datos, se seleccionaron el *Medical Literature Analysis and Retrieval System Online* (MEDLINE), la base de datos *Excerpta Medica* (Embase), la *Virtual Health Library* (VHL), *Latin American and Caribbean Literature in Health Sciences* (LILACS) y Google *Scholar* (para literatura gris), ya que son ampliamente reconocidos en el campo de la salud. El objetivo era analizar la producción científica sobre el tema propuesto en contextos nacionales e internacionales.

La búsqueda avanzada de los artículos se llevó a cabo en enero de 2024, simultáneamente en las cinco bases de datos. La selección y evaluación de los estudios se realizó de forma independiente y sin supervisión por dos revisores, y cualquier desacuerdo se resolvió por consenso con un tercer revisor^25^. Para seleccionar los estudios iniciales y excluir duplicados, los revisores leen los títulos y resúmenes utilizando el gestor de referencias en línea Rayyan. La selección realizada por cada revisor se ordenaba sistemáticamente con el apoyo del *software* y, posteriormente, se comparaba entre sí para la validación de la muestra final^26^.

Las palabras clave se seleccionaron de los Descriptores de Ciencias de la Salud (DeCS), a saber: “Tecnología Educativa”, “Health Education”, “Persona con Discapacidad”, “Disabled Persons”, “Educación para la Salud” y “Health Education”, utilizando los operadores booleanos OR y AND. Se combinaron de diferentes maneras, como: *Tecnología Educativa* OR *Educación para la Salud* AND *Persona con discapacidades*; *Tecnología Educativa AND* educación para la salud AND *Persona con discapacidad*, en portugués e inglés. Los resultados se muestran en la Figura 1 a continuación:

**Figura 1:** Tabla de descripción de operadores booleanos para la búsqueda en bases. Campina Grande, PB, Brasil, 2024

Mecanismo de búsqueda	Términos de investigación	Citas recuperadas
MEDLINE	*(“Educational Technology”\[MeSH Terms] OR “educational technology”\[tiab] OR “tecnologia educacional”\[tiab]) AND (“Health Education”\[MeSH Terms] OR “health education”\[tiab] OR “educação em saúde”\[tiab]) AND (“Disabled Persons”\[MeSH Terms] OR “people with disabilities”\[tiab] OR “pessoa com deficiência”\[tiab])*	78
Embase	*(‘educational technology’/exp OR ‘educational technology’\:ti,ab OR ‘tecnologia educacional’\:ti,ab) AND (‘health education’/exp OR ‘health education’\:ti,ab OR ‘educação em saúde’\:ti,ab) AND (‘disabled person’/exp OR ‘people with disabilities’\:ti,ab OR ‘pessoa com deficiência’\:ti,ab)*	57
VHL*	*(“Tecnologia Educacional” OR “tecnologia educacional” OR “Educational Technology”) AND (“Educação em Saúde” OR “Health Education”) AND (“Pessoas com Deficiência” OR “pessoa com deficiência” OR “People with Disabilities”)*	23
LILACS	*(“Tecnologia Educacional” OR “Educational Technology”) AND (“Educação em Saúde” OR “Health Education”) AND (“Pessoas com Deficiência” OR “People with Disabilities”)*	29
Literatura gris	*(“tecnologia educacional” OR “educational technology”) AND (“educação em saúde” OR “health education”) AND (“pessoa com deficiência” OR “people with disabilities”)*	13
**Total**		200

*VHL = *Virtual Health Library*

### Criterios de elegibilidad

La selección de los estudios analizados se basó en los siguientes criterios de inclusión: abordar el tema en curso; texto disponible en su totalidad y ser escrito o traducido al portugués, inglés y/o español. Los criterios de exclusión eran: textos incompletos o textos con acceso indisponible; repetición del mismo artículo en más de una base de datos; falta de relación con el objeto de estudio. No se consideró reducir el tiempo de investigación para recuperar el mayor número posible de estudios.

### Tratamiento y análisis de datos

Las directrices de la estrategia PRISMA-ScR para revisiones de alcance^27^ se utilizaron para representar gráficamente el procedimiento de selección de los estudios, tal como se muestra en el diagrama de flujo del procedimiento de búsqueda y selección de los estudios elegibles en la revisión (Figura 2).

En su edición de noviembre de 2024, el JBI *Manual for Evidence Synthesis* refuerza la importancia de abordar la calidad de la evidencia incluso en las revisiones de alcance. Se reconoce que, aunque este tipo de revisión no pretende evaluar críticamente todos los estudios incluidos, se recomienda una descripción sistemática de posibles sesgos, limitaciones metodológicas y heterogeneidad de la evidencia^28^.

Según el manual, el nivel general de confianza y las características de los estudios (por ejemplo, riesgo de sesgo, imprecisión, inconsistencia) deberían clasificarse al menos, para que el lector comprenda el grado de robustez de las conclusiones extraídas en la síntesis. Este enfoque contribuye a la transparencia metodológica y permite identificar lagunas de conocimiento, guiando futuras revisiones con metodologías más rigurosas o específicas^28^.

## Resultados

Los procedimientos metodológicos utilizados en la composición del corpus de investigación se representaron gráficamente mediante un diagrama de flujo, elaborado a partir de las directrices de la estrategia PRISMA-ScR para las revisiones de alcance, como se muestra en la Figura 2 a continuación:

**Figura 2:** Diagrama de flujo PRISMA-ScR utilizado para la identificación y selección de estudios. Campina Grande, PB, Brasil, 2024

*VHL = *Virtual Health Library*

Inicialmente, con la aplicación de filtros (texto completo; idioma: portugués, inglés y español) en las bases de datos seleccionadas, se identificaron 200 artículos mediante las búsquedas de esta revisión; los datos de estos artículos se importaron a la plataforma Rayyan. El *software* Rayyan resume automáticamente los años de publicación de los estudios importados; Con esto, se supo que la primera publicación con estos descriptores tuvo lugar en 2015, sin embargo, entre 2019 y 2023, las publicaciones aumentaron; En 2019 se encontraron 49 publicaciones, en 2020 51, en 2021 y 2022 hubo 34 publicaciones cada año y, en 2023, el número de publicaciones encontradas relacionadas con el tema propuesto descendió a 24. Esta información fue extraída de Rayyan.

La muestra final consistió en ocho estudios, siete de los cuales fueron publicados en revistas científicas y una tesis doctoral. En cuanto al país de origen, siete se realizaron en Brasil y uno en Rumanía, publicado en inglés. El periodo de publicación varió entre 2015 y 2022.

Las poblaciones objetivo incluían diferentes tipos de discapacidad: tres estudios se centraron en personas con discapacidad auditiva, tres en personas con discapacidad visual, uno en personas con discapacidad intelectual y uno en personas con deterioro cognitivo. En cuanto a las tecnologías utilizadas, se identificaron tecnologías educativas digitales e impresas (como folletos y cursos online accesibles), tecnologías de asistencia (*software*, avatares, recursos de accesibilidad digital) y tecnologías innovadoras (*exergames*). Los temas abarcaban desde la salud bucodental, la salud sexual y reproductiva, y la prevención del abuso sexual hasta la hipertensión, la salud mamaria y la actividad física.

En cuanto a la autoría, la mayoría de las producciones fueron realizadas por investigadores en enfermería, con participación colaborativa de otras áreas en casos específicos, como educación física, informática y educación. En general, todos los estudios reportaron resultados positivos en términos de validez, accesibilidad, aceptabilidad y viabilidad de las tecnologías, aunque limitados por tamaños de muestra pequeños, ausencia de grupo de control y corta duración del seguimiento. La Figura 3 a continuación muestra la caracterización de los estudios incluidos.

**Figura 3:** Descripción de los estudios incluidos. Campina Grande, PB, Brasil, 2024

Nº	Estudio (autor/año)	Tipo de publicación/País	Populación objetivo/Discapacidad	Tecnología/ Clasificación/Tema	Principales hallazgos	Categoría profesional de autores
1	Nóbrega, et al. (2021)	Artículo/Brasil	Jóvenes con discapacidad intelectual	Tecnología educativa validada/Kit educativo con libro, folleto de apoyo, muñecos y vídeo explicativo, dirigido a la prevención de violencia sexual.	Alta validez de contenido (IVC*≈0,99); potencial para prevención, pero no probado en el público objetivo.	Enfermería
2	Oliveira, et al. (2020)	Artículo/Brasil	Personas con discapacidad auditiva (usuarios de Libras)	Tecnología educativa/Panel ilustrativo para facilitar la comunicación en salud entre enfermeros y usuarios.	El recurso en *Libras* favorece la interacción entre enfermeros y personas sordas; evidencia de aceptabilidad, sin medir la eficacia.	Enfermería
3	Áfio, et al. (2016)	Artículo/Brasil	Personas con discapacidad auditiva	Tecnología asistiva/Curso online “Educación en Salud Sexual y Reproductiva: uso de preservativos”	El análisis automático indicó conformidad técnica; la ausencia de pruebas con usuarios limitó la aplicabilidad.	Enfermería
4	Carvalho, Pagliuca & Fernandes (2015)	Artículo/Brasil	Personas con discapacidad visual (ciegas)	Tecnología educativa/Curso online en salud mamaria.	Validación inicial positiva del contenido y la accesibilidad; muestra pequeña	Enfermería
5	Carvalho, et al. (2018)	Artículo/Brasil	Personas con discapacidad visual	Tecnología educativa/Curso online sobre hipertensión arterial.	Curso accesible y considerado satisfactorio; sin evaluación de impacto clínico.	Enfermería
6	Marques (2017)	Tesis/Brasil	Personas con discapacidad visual (ciegas)	Tecnología educativa/Folleto virtual para la prevención de la violencia sexual.	Validación metodológica robusta; aceptabilidad positiva; muestra piloto pequeña.	Enfermería
7	Vaghetti, et al. (2022)	Resumen ampliado en los anales/Brasil	Adultos con deterioro cognitivo	Tecnología innovadora/*Exergames*/Movimientos corporales requeridos por el juego (*gameplay)* que se utiliza que promueven la actividad física.	*Exergames* **promovieron la socialización y la autonomía; viabilidad confirmada; ausencia de grupo de control.**	Educación Física y Enfermería (colaboración)
8	Chiriac, et al. (2021)	Artículo/Rumanía	Personas con discapacidad auditiva (sordas)	Tecnología asistiva/Avatares y vídeos para salud bucodental.	Vídeos más sencillos y baratos; avatares más complejos; ambos favorecían la comunicación.	Equipo multidisciplinar (Educación, Salud y Computación)

*IVC= Índice de Validez de Contenido

El conjunto analizado está compuesto predominantemente por estudios metodológicos sobre el desarrollo y validación de tecnologías educativas y asistenciales, informes de experiencia y estudios de intervención exploratoria, centrados en diferentes tipos de discapacidades (intelectuales, visuales, auditivas y cognitivas). La mayoría de los estudios se desarrollaron en Brasil, con la excepción de uno realizado en Rumanía, que refleja una producción concentrada en contextos latinoamericanos.

Los principales tipos de tecnologías identificadas fueron: tecnologías educativas digitales e impresas: folletos virtuales y cursos online accesibles, desarrollados para personas ciegas, y que abordaban temas como la salud sexual, la prevención de la violencia y la hipertensión. Tecnologías de asistencia: *software* de accesibilidad (lectores de pantalla, evaluación automática de accesibilidad) y adaptaciones para la comunicación en Libras. Tecnologías innovadoras: *exergames* y avatares, utilizados tanto para promover la actividad física en personas con discapacidades cognitivas como para facilitar la comunicación en salud bucal para personas sordas.

En cuanto a los principales hallazgos, los estudios indicaron que las tecnologías validadas presentaban un alto acuerdo entre expertos en cuanto a contenido y apariencia, como en un estudio^11^ en el que la tecnología para la prevención de la violencia sexual en jóvenes con discapacidad intelectual obtuvo un índice de validez de contenido cercano a 1,0. Se encontró que los recursos educativos accesibles para personas ciegas, como los manuales virtuales y los cursos en línea, eran viables, comprensibles y adecuados, aunque se analizaron en muestras pequeñas y generalmente sin evaluación longitudinal de impacto.

El uso de *exergames* demostró viabilidad pedagógica y potencial para promover la socialización, la autonomía y las habilidades motoras, a pesar de la ausencia de grupos de control y la heterogeneidad de los tipos de juegos aplicados. El uso de avatares y vídeos en Libras indicó beneficios en la comunicación sanitaria para personas sordas, pero los estudios se limitaron a análisis técnicos o pilotos con pocos participantes.

En cuanto a la evaluación de la calidad de la evidencia, de acuerdo con las directrices del Manual de Síntesis de Evidencia del JBI^28^, se realizó una descripción sistemática de posibles sesgos, limitaciones metodológicas y heterogeneidad. En general: los estudios presentaron muestras pequeñas, a menudo seleccionadas por conveniencia, lo que genera un riesgo de sesgo de selección y limita la generalización. Muchos estudios se centraron únicamente en validar el contenido y la aparición con expertos, sin probar la efectividad en poblaciones de usuarios, restringiendo la solidez de la evidencia para evaluar el impacto en la salud.

La heterogeneidad entre tecnologías (folletos, cursos, *software*, *exergames*, avatares) y entre resultados (validez del contenido, aceptabilidad, cumplimiento técnico, viabilidad) hace imposible comparaciones directas o metaanálisis, lo que indica la necesidad de una síntesis narrativa. La calidad global de la evidencia se calificó como moderada en validez y aceptabilidad y baja en eficacia clínica o cambio de comportamiento, ya que ninguno de los estudios empleó diseños robustos como ensayos controlados aleatorizados o seguimientos longitudinales.

Estos hallazgos demuestran que, aunque la producción existente es limitada en términos de rigor metodológico, los estudios proporcionan subvenciones relevantes para la práctica de la educación sanitaria y para el desarrollo de tecnologías sanitarias inclusivas, especialmente en lo que respecta a la viabilidad, accesibilidad y adecuación cultural de los recursos. El cuadro construido (Figura 4), de acuerdo con el Manual del JBI, presenta en detalle los posibles sesgos, limitaciones metodológicas y la calidad atribuida a cada estudio incluido, sirviendo como referencia complementaria a la síntesis narrativa.

**Figura 4:** Descripción de los estudios incluidos. Campina Grande, PB, Brasil, 2024

Nº	Tipo de estudio	Sesgos potenciales	Limitaciones metodológicas	Heterogeneidad de las evidencias	Calidad de la evidencia - con justificación
1	Estudio metodológico, validación de contenido/apariencia por parte de los jueces	Sesgo de selección: especialistas (expertos) en conveniencia; Sesgo de evaluador disciplinario.	No hubo validación con usuarios finales; enfoque en especialistas.	Evidencia sólida solo en validez del contenido.	Moderada (validez de contenido), Baja (efectividad). Justificación: IVC*=0,99 indica una excelente concordancia, pero sin pruebas en jóvenes con DI^†^.
2	Informe de experiencia en Educación Permanente	Sesgo de los autores en la información; selección por conveniencia de enfermeros.	No hay mediciones estandarizadas ni grupos de comparación.	Pruebas de aceptabilidad; no mide la efectividad.	Baja. Justificación: muestra barreras e importancia de los *Libras*, pero sin rigor metodológico.
3	Evaluación técnica de la conformidad	Sesgo de medición por análisis automático; sin usuarios.	No se ha probado a gran escala con personas sordas.	Homogéneo en criterios técnicos; poco comparable a los estudios clínicos.	Moderado (cumplimiento), bajo (eficacia). Justificación: Útil para estándares técnicos, pero no garantiza la usabilidad.
4	Estudio metodológico/validación inicial	Muestra demasiado pequeña; ajustes realizados según sugerencias.	Sin evaluación de impacto en el comportamiento o el conocimiento.	Alta heterogeneidad temática.	Moderada (validación inicial), Baja (eficacia). Justificación: validación inicial robusta, pero pequeña escala.
5	Estudio metodológico (modelo Falkembach)	Sesgo del proyecto (evaluados por desarrolladores); muestra pequeña.	No evalúa los resultados clínicos (por ejemplo, control de la presión arterial).	Alta heterogeneidad entre cursos/temas.	Moderada (accesibilidad), baja (impacto clínico). Justificación: estructurada, pero sin efectividad en salud.
6	Estudio metodológico, revisión, GF^‡^, validación y piloto (n=22)	Muestra piloto pequeña; sesgo de respuesta social.	Sin seguimiento longitudinal; ausencia de resultados conductuales.	Evidencia consistente de aceptabilidad; pocos estudios comparables.	Moderada (validación/aceptabilidad), Bajo (impacto). Justificación: robusta, pero limitada por el tamaño de la muestra.
7	Intervención exploratoria con 26 participantes	Muestra pequeña; sesgo de observador.	Sin grupo de control; varias consolas/juegos.	Heterogeneidad elevada entre juegos y resultados.	Baja-Moderada. Justificación: muestra viabilidad, pero no eficacia sostenida.
8	Estudio comparativo técnico	Sesgo de implementación (diferencia de coste/tiempo); pocos participantes.	Resultados iniciales; sin medidas robustas de aprendizaje.	Evidencia técnica específica.	Baja (eficacia), Moderada (evaluación técnica). Justificación: útil para decisiones técnicas, pero no muestra impacto educativo.

*IVC = Índice de Validez de Contenido; ^†^DI = Discapacidad Intelectual; ^‡^GF = Grupo Focal

## Discusión

La presente revisión de alcance identificó ocho estudios, en su mayoría metodológicos, centrados en el desarrollo y validación de tecnologías educativas y asistenciales. Esta predominancia refleja la naturaleza aún emergente del campo, en el que la producción científica se centra en proponer y probar recursos innovadores, en lugar de en estudios de impacto o revisiones sistemáticas. Para garantizar la claridad terminológica, es importante distinguir las tecnologías educativas (TE), asociadas a los procesos de enseñanza-aprendizaje, de las tecnologías asistenciales (TA), destinadas a promover la accesibilidad, la autonomía y la inclusión de las personas con discapacidad^5^ ^-^ ^6^. Esta diferenciación guió el análisis de los hallazgos y evita términos que se solapuen.

Los datos se organizaron según los tipos de tecnologías identificadas (educativas, asistenciales, digitales innovadoras), sus contextos de aplicación (salud bucal, salud sexual y reproductiva, prevención de enfermedades, actividad física y comunicación en salud) y los grupos poblacionales a los que se sirvió (personas con discapacidades auditivas, visuales, intelectuales y cognitivas). Esta sistematización nos permitió comprender la diversidad de enfoques y la convergencia de resultados en torno a aspectos comunes, como la validez, la viabilidad, la accesibilidad y la aceptación de las tecnologías.

En el ámbito de la salud sexual y reproductiva, destaca el estudio que validó una tecnología educativa destinada a prevenir la violencia sexual en jóvenes con discapacidad intelectual^11^, poniendo de manifiesto un tema que a menudo se silencia. De forma complementaria, otro autor desarrolló un folleto virtual para personas con discapacidad visual, centrado también en la prevención de la violencia sexual^29^, reforzando el potencial de las tecnologías educativas para abordar cuestiones sensibles y desatendidas.

La comunicación en salud fue otro eje central, con énfasis en los autores que desarrollaron un TE basado en Libras para facilitar la interacción entre enfermeros y personas sordas^10^, revelando que las barreras lingüísticas siguen siendo obstáculos para la plena inclusión en los servicios de salud. En este mismo campo, otros autores han aplicado el instrumento *Automatic System for Evaluating Software Accessibility* (ASES) al contenido digital, identificando el cumplimiento técnico, pero subrayando la importancia de evaluar también la experiencia real de los usuarios sordos^30^.

El uso de modelos validados de diseño instruccional resultó estratégico para la construcción de tecnologías accesibles. Un ejemplo de esto son los cursos online sobre salud mamaria^31^ e hipertensión^32^, ambos desarrollados en la Universidad Federal de Ceará. Estos estudios utilizaron el modelo de Falkembach y demostraron que metodologías consistentes pueden garantizar calidad, accesibilidad y seguridad en el desarrollo de recursos digitales para personas ciegas.

En el ámbito de la salud bucodental, destaca una contribución internacional. Un estudio realizado en Rumanía comparó el uso de avatares y vídeos en aplicaciones para personas sordas, mostrando que ambos recursos favorecían la comunicación en salud bucodental, aunque con diferencias en coste y complejidad^33^.

Finalmente, el uso de ejercicios en programas de actividad física para personas con deterioro cognitivo^34^ reveló beneficios relacionados con la socialización, el aumento de habilidades motoras y la autonomía, demostrando ser una alternativa viable y de bajo coste para la promoción de la salud en este público.

En general, los hallazgos demuestran que tanto las TE como las TA emergen como recursos valiosos para la promoción de la salud inclusiva, contribuyendo a la educación en salud, la prevención de enfermedades, la rehabilitación y la inclusión social. La síntesis muestra que diferentes áreas (salud sexual y reproductiva, salud bucal, comunicación en salud y actividad física) pueden beneficiarse de tecnologías cuando se desarrollan y aplican de manera inclusiva.

Aun así, cabe señalar que la mayoría de los estudios se centraron en las primeras etapas del desarrollo y la validación, sin avanzar hacia análisis de efectividad en contextos reales o a gran escala. Vale la pena reforzar la importancia de futuros estudios para ampliar las muestras, incluir un grupo de control y evaluar el impacto de las tecnologías en los resultados clínicos y sociales. Por último, cabe señalar que la pandemia de COVID-19, aunque ha impuesto desafíos sanitarios, también ha impulsado la adopción de herramientas digitales inclusivas, allanando el camino para nuevos estudios y prácticas innovadoras, pero sin eliminar las brechas de acceso ni la necesidad de políticas públicas que garanticen la sostenibilidad y la equidad.

## Conclusión

Las tecnologías educativas digitales e impresas, las tecnologías de asistencia y las tecnologías innovadoras abarcan diferentes formatos (cursos online accesibles, folletos digitales, recursos en Libras, aplicaciones, avatares y ejercicios) y estaban dirigidas a diversos públicos, incluyendo personas con discapacidades auditivas, visuales, intelectuales y cognitivas. En general, los estudios demostraron que las tecnologías desarrolladas tienen buena aceptación, validez del contenido, accesibilidad y viabilidad, contribuyendo al empoderamiento, inclusión y fortalecimiento de la práctica profesional en salud, especialmente en enfermería.

A pesar de la diversidad de propuestas, se observó que la mayor parte de la investigación se centraba en las etapas de desarrollo y validación, sin avanzar a análisis de efectividad a gran escala. Además, predominó los estudios metodológicos e intervenciones exploratorias. Esta característica apunta tanto a la relevancia del campo en expansión como a la necesidad de investigaciones más sólidas, con muestras más amplias, seguimiento longitudinal y comparaciones controladas, para fortalecer la calidad de la evidencia.

Por lo tanto, se considera que este estudio ha alcanzado su objetivo, demostrando que las tecnologías educativas de salud dirigidas a personas con discapacidad son herramientas relevantes para promover la inclusión y la autonomía. Sin embargo, la revisión también pone de relieve carencias importantes, como la limitación de las bases de datos consultadas, la concentración geográfica de la producción científica y la escasez de estudios que evalúen los impactos concretos de estas tecnologías. Se recomienda que futuras investigaciones amplíen las estrategias de búsqueda, diversifiquen los contextos de investigación y avancen en los análisis de eficacia, con el fin de consolidar la contribución de las tecnologías educativas a la salud inclusiva.

## Data Availability

Todos los datos generados o analizados durante este estudio están incluidos en este artículo publicado.
